# Economical and preferred walking speed using body weight support apparatus with a spring-like characteristics

**DOI:** 10.1186/s13102-021-00336-7

**Published:** 2021-09-06

**Authors:** Daijiro Abe, Shunsuke Sakata, Kiyotaka Motoyama, Naoki Toyota, Hidetsugu Nishizono, Masahiro Horiuchi

**Affiliations:** 1 Center for Health and Sports Science, Kyushu Sangyo University, 2-3-1 Matsukadai, Higashi- ku, Fukuoka, 813-8503 Japan; 2 Department of Management and Information, Faculty of Commerce, Yokohama College of Commerce, Yokohama, Kanagawa Japan; 3 Department of Sport Science and Health, Faculty of Human Sciences, Kyushu Sangyo University, Fukuoka, Japan; 4 Division of Human Environmental Science, Mt. Fuji Research Institute, Fujiyoshida, Yamanashi Japan

**Keywords:** Locomotion, Gait, Walking economy, Hypogravity, Optimal speed, Reduced gravity

## Abstract

**Background:**

A specific walking speed minimizing the U-shaped relationship between energy cost of transport per unit distance (CoT) and speed is called economical speed (ES). To investigate the effects of reduced body weight on the ES, we installed a body weight support (BWS) apparatus with a spring-like characteristics. We also examined whether the 'calculated' ES was equivalent to the 'preferred' walking speed (PWS) with 30% BWS.

**Methods:**

We measured oxygen uptake and carbon dioxide output to calculate CoT values at seven treadmill walking speeds (0.67–2.00 m s^− 1^) in 40 healthy young males under normal walking (NW) and BWS. The PWS was determined under both conditions on a different day.

**Results:**

A spring-like behavior of our BWS apparatus reduced the CoT values at 1.56, 1.78, and 2.00 m s^− 1^. The ES with BWS (1.61 ± 0.11 m s^− 1^) was faster than NW condition (1.39 ± 0.06 m s^− 1^). A Bland-Altman analysis indicated that there were no systematic biases between ES and PWS in both conditions.

**Conclusions:**

The use of BWS apparatus with a spring-like behavior reduced the CoT values at faster walking speeds, resulting in the faster ES with 30% BWS compared to NW. Since the ES was equivalent to the PWS in both conditions, the PWS could be mainly determined by the metabolic minimization in healthy young males. This result also derives that the PWS can be a substitutable index of the individual ES in these populations.

## Background

There is a U-shaped relationship between the energy cost of transport per unit distance (CoT; J kg^− 1^ m^− 1^) and walking speed (m s^− 1^) [1]. This U-shaped CoT-speed relationship in walking provides a specific walking speed minimizing the CoT in each individual, which is called the optimal speed [2] or economical speed (ES) [3–6]. The ES or a speed at which the CoT becomes minimal is inherent in each individual under normoxia or moderate hypoxia on a shallow gradient [4–6], in the elderly participants who received long-term training [7], and in obese adolescents with a successful reduction of body fat [8]. On the contrary, the ES becomes slower due to load carriage [9], obesity [10–12], and increased body weight (BW) during pregnancy [2]. Collectively, these results suggest that the ES becomes slower when BW is increased.

BW support (BWS) is available to provide an acute experimental reduction of BW during walking, and it reduces the CoT in human locomotion [13, 14]. The oxygen consumption was much lower with BWS at heavy exercise intensities, but not so much at light and/or moderate exercise intensities [13], assuming that the effect of BWS on the U-shaped CoT-speed relationship in walking could be greater at faster speeds than slower speeds. These assumptions suggested that a rightward (faster) shift of the U-shaped CoT-speed relationship would be observed with BWS compared to normal walking (NW). Since a rightward shift of the U-shaped CoT-speed relationship provides a faster ES [15], we hypothesized that the ES would be faster with BWS compared to NW conditions.

In relation to the physiological and clinical significances of the ES, many studies have reported that the preferred walking speed (PWS) was almost equivalent or close to the ES in able-bodied individuals regardless of obesity [10–12], pregnancy [2], and sex [10]. In contrast, individuals who have undergone heart surgery or a lower limb amputation and prosthesis users prefer a slower PWS than their 'calculated' ES [3]. These results suggested that the able-bodied individuals selected their PWS on the basis of minimized CoT during walking. However, calculating the ES is best done by measuring the CoT values at 5–8 different speeds [3–6, 8–12, 15, 16], indicating that 20–30 min is required to determine each individual ES. This is presumably fine for a small number of fit individuals, but it could be hard for individuals whose physical fitness is poor. If the PWS is equivalent to the ES, then the ES of individuals whose physical fitness is poor could be roughly predicted with a significant reduction of their physical strain. We also hypothesized that the PWS could be equivalent to the ES irrespective of the use of BWS if minimizing metabolic cost is the primary factor explaining the PWS. However, the ES and PWS have not yet been tested with BWS in a relatively large number of healthy young participants. Thus, the purpose of this study was to compare the ES and PWS under the BWS and NW conditions to test the above hypotheses in this study.

## Methods

### Participants

Forty healthy young males including 13 recently tested participants [17] were involved in this study. They are recreationally trained with some previous walking experiences on a treadmill. It has been reported that there is a sex difference in the U-shaped CoT-speed relationship in walking [8, 18], so we recruited male participants only. Their mean age, stature, and BW were 19.7 ± 0.8 years, 1.70 ± 0.06 m, 62.8 ± 8.7 kg, respectively [mean ± standard deviation (SD)]. BW was measured before testing the BWS condition. In accordance with the Declaration of Helsinki, a written informed consent was obtained from all participants after they were provided with information about the purposes, experimental protocols, and possible risks of this study. This study was reviewed and approved by the ethical committee established in Kyushu Sangyo University before starting the data collection (H28-0001-1).

### Body weight support (BWS)

**Fig. 1:**
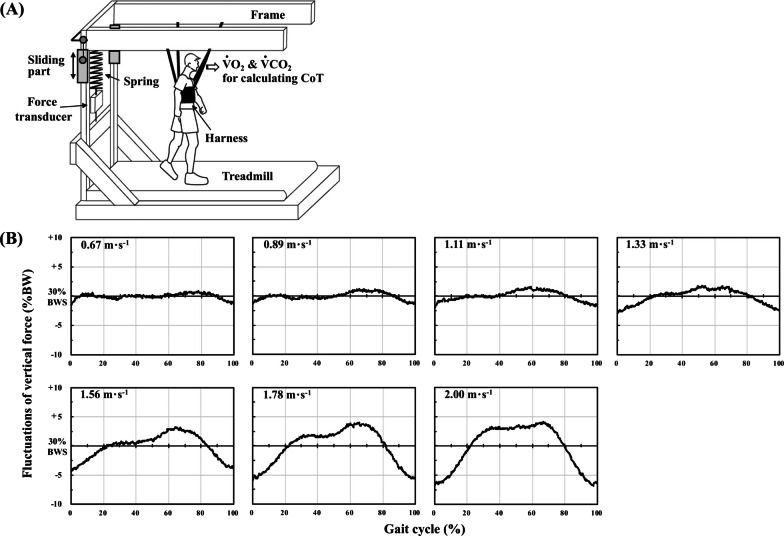
Body weight support (BWS) apparatus and its functional characteristics. **a** A schematic illustration of the participants walking with a custom-made BWS apparatus. This setting ensured that the BWS apparatus applied vertical force without disturbing leg swing motion. VO_2_ and VCO_2_ are oxygen uptake and carbon dioxide output, respectively. CoT is the energy cost of transport per unit distance. **b** Fluctuations of the vertical force in four representative participants from 0.67 to 2.00 m s^− 1^. Five steps in each participant were time-aligned into percentage of gait cycle, and those data were overlapped at each speed. The frame position becomes the lowest at the heel contact, so the timing at the lowest vertical force was regarded as 0% gait cycle. %BW means percentage of supported body weight.

A body suspension apparatus that lifts the participants’ torso by an elastic harness was installed surrounding a motor-driven treadmill (Fig. 1a; Well Load 200E, Takei Scientific Instruments Co., Ltd., Niigata, Japan) [17]. A force transducer (TSA-110, Takei Scientific Instruments Co., Ltd., Japan) was situated between a controller and a spring (30 cm free length with a spring constant of 5.7 kg cm^− 1^). In the preliminary testing, most of the participants were uncomfortable at the inguinal region if more than 30% of their BW was supported. Thus, we chose 30% of their BW by the spring segments. This setting allowed the participants to swing their legs normally.

### Protocols and determination of economical speed (ES)

All participants continuously walked on the treadmill at seven walking speeds (0.67, 0.89, 1.11, 1.33, 1.56, 1.78, and 2.00 m s^− 1^) in the order from the slowest (0.67 m s^− 1^) to the fastest (2.00 m s^− 1^) speed on the level (0%) gradient for 4 min at each walking speed. The order of NW and BWS conditions were randomized, and the latter condition was tested 5–7 days later after testing the former condition. At any walking speed, the participants were asked to select their preferred step frequencies. They wore underwear, short sleeve T-shirts, running shorts, socks, and same shoes with different sizes. Total weight of these clothes ranged from 0.3 to 0.4 kg. Air temperature of the experimental room was maintained from 19 to 20 °C. Oxygen uptake (VO_2_; mL kg^− 1^ s^− 1^) and carbon dioxide output (VCO_2_; mL kg^− 1^ s^− 1^) were continuously measured with a computerized breath-by-breath system (AE-310S, Minato Ltd, Japan). Calibration of the gas analyzers was conducted before each measurement with well-known gas concentrations (O_2_ 15.22%, CO_2_ 5.17%, and N_2_ 79.61%) and room air. An average VO_2_ and VCO_2_ for the final 2 min at each speed was provided to calculate the CoT values with a following equation [19].

1 $$\begin{aligned} & {\text{CoT}}\;\left( {{\text{J}}\;{\text{kg}}^{{ - 1}} \;{\text{m}}^{{ - 1}} } \right) \\ & \quad = \frac{{4.186~ \times ~\left( {3.869 \times {\text{VO}}2~ + ~1.195 \times {\text{VCO}}2} \right)}}{{speed}} \\ \end{aligned}$$

The CoT values were compared at each walking speed between conditions. A U-shaped relationship between CoT values and walking speeds in each participant was approximated with a quadratic equation [4–6]:

2 $${\text{CoT}}\;\left( s \right) = {\text{a}}{\cdot} speed^{2} + {\text{b}}{\cdot }speed + {\text{c}}$$

where the coefficients a, b, and c are determined by the least squares regression with CoT values obtained from seven walking speeds. The 'calculated' ES, at which the U-shaped CoT-speed relationship in walking becomes minimal, can be obtained when a differential function of the Eq. 2 (CoT’(*s*) = 2a *speed* + b) is zero [4–6]. Thus, the ES in each participant was determined with a following equation:

3 $${\text{ES}}\;\left( {{\text{m}}\;{\text{s}}^{{ - 1}} } \right)~ = ~\frac{{\left| { - b} \right|}}{{2a}}$$

The use of either net CoT values (absolute minus standing energy cost) or gross (absolute) CoT values may be a matter of some controversy [2, 4]. A previous study argued that the standing energy cost represented different physiological states than the dynamic state of steady-speed walking [2]. The ES calculated by the net CoT values was significantly slower than that calculated by using the gross CoT values [2]. Several studies observed that healthy people prefer walking speeds at or near the speed associated with their minimum gross CoT [2, 3, 8, 10, 11, 16], so we used the gross CoT values in this study. To make potential comparisons with other studies, we report the mean standing energy cost was 97.8 ± 12.0 J kg^− 1^ min^− 1^ (1.56 ± 0.19 watts kg^− 1^).

### Determination of preferred walking speed (PWS)

The PWS was determined within a few days after the metabolic measurements under the BWS and NW conditions. The participant warmed up on the treadmill at or near his 'calculated' ES for 1 min and then rested while seated on a chair. After the warming up session, the participant started walking on the treadmill in a ramp manner under the control of external computer (4.63 × 10^− 3^ m s^− 2^ = 1 km h^− 1^ per minute) from 0.56 m s^− 1^. When the participant initially chose his PWS, he held that treadmill speed for a while. And then the voluntarily modified the treadmill speed up or down by 2.78 × 10^− 2^ m s^− 1^ (0.1 km h^− 1^) to finally determine his PWS [16]. During the PWS determination protocol, treadmill monitor panel was covered with a corrugated board not to present the treadmill speed to the participants, indicating that a hysteresis effect and tester’s behavior (e.g. manual operation of the treadmill speed on the monitor) could not influence the individual PWS. The order of the BWS and NW conditions was randomized.

### Statistical analysis

The ESs observed under the BWS and NW conditions were compared with a paired two-tailed *t*-test. The CoT values as well as observed ES and PWS with BWS and NW were compared with a two-way repeated measures analysis of variance (ANOVA) within-participants using online software (ANOVA 4). If a significant *F* values was obtained, Ryan’s post hoc test was further applied to the appropriate data sets to detect significant mean differences; its statistical power has been reported to be equivalent to Tukey’s post hoc test [20], and it can be used regardless of the data distribution [20]. A Bland–Altman plot was applied to evaluate whether each data set involved systematic bias [21], which consists of 'fixed' bias and 'proportional' bias. If 95% confidence interval (CI_95%_) of the individual PWS–ES differences included zero, there was no systematic bias between ES and PWS in each condition. The CI_95%_ was determined by a following equation

4 $${\text{CI}}_{{95\% }} = d_{{\text{m}}} \pm t_{{39}} \cdot\surd SD_{{\text{d}}} \cdot{\text{n}}^{{ - 1}}$$

where *d*_m_ is the mean of the individual PWS–ES differences, *t*_39_ is the *t*-distribution value with a degree of freedom of 39, *SD*_d_ is the standard deviation of the individual PWS–ES differences, and n is the total number of the participants. To evaluate whether the data set involved proportional bias, a correlation analysis was conducted between individual PWS–ES differences and individual average of the ES and PWS. If there was a significant correlation, there was a proportional bias between ES and PWS. The upper and lower limits of agreement were determined using a following equation:

5 $${\text{Upper}}\;{\text{and}}\;{\text{lower}}\;{\text{limits}}\;{\text{of}}\;{\text{agreement}} = d_{{\text{m}}} \pm 1.96{\cdot}SD_{{\text{d}}}$$

Statistical significance was set at *p* < 0.05. All data were presented as mean ± SD.

## Results

**Fig. 2:**
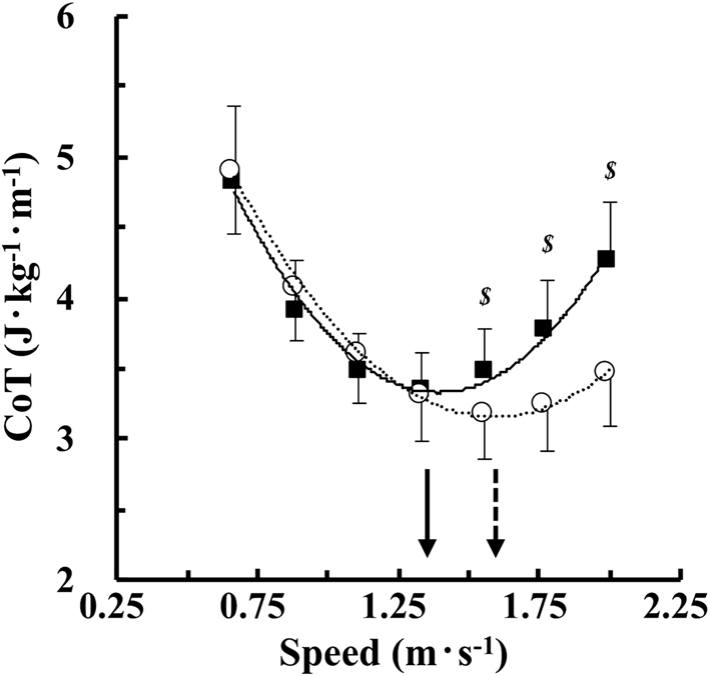
CoT-speed relationships under normal walking (NW) and BWS. The CoT values were lower with BWS (open circles; *y* = 1.987*x*^2^ − 6.325*x* + 8.191, *r* = 0.998, n = 40) than NW (black filled squares; *y* = 2.709*x*^2^ − 7.533*x* + 8.569, *r* = 0.990, n = 40) at faster than 1.56 m s^− 1^. A faster economical speed (ES) was observed with BWS than NW due to a rightward (faster) shift of the U-shaped CoT-speed relationship. Broken and solid arrows indicate the average ES with BWS and NW, respectively. ^*$*^ *p* < 0.001 between BWS and NW at each walking speed. Values are mean ± standard deviation (SD).

**Fig. 3:**
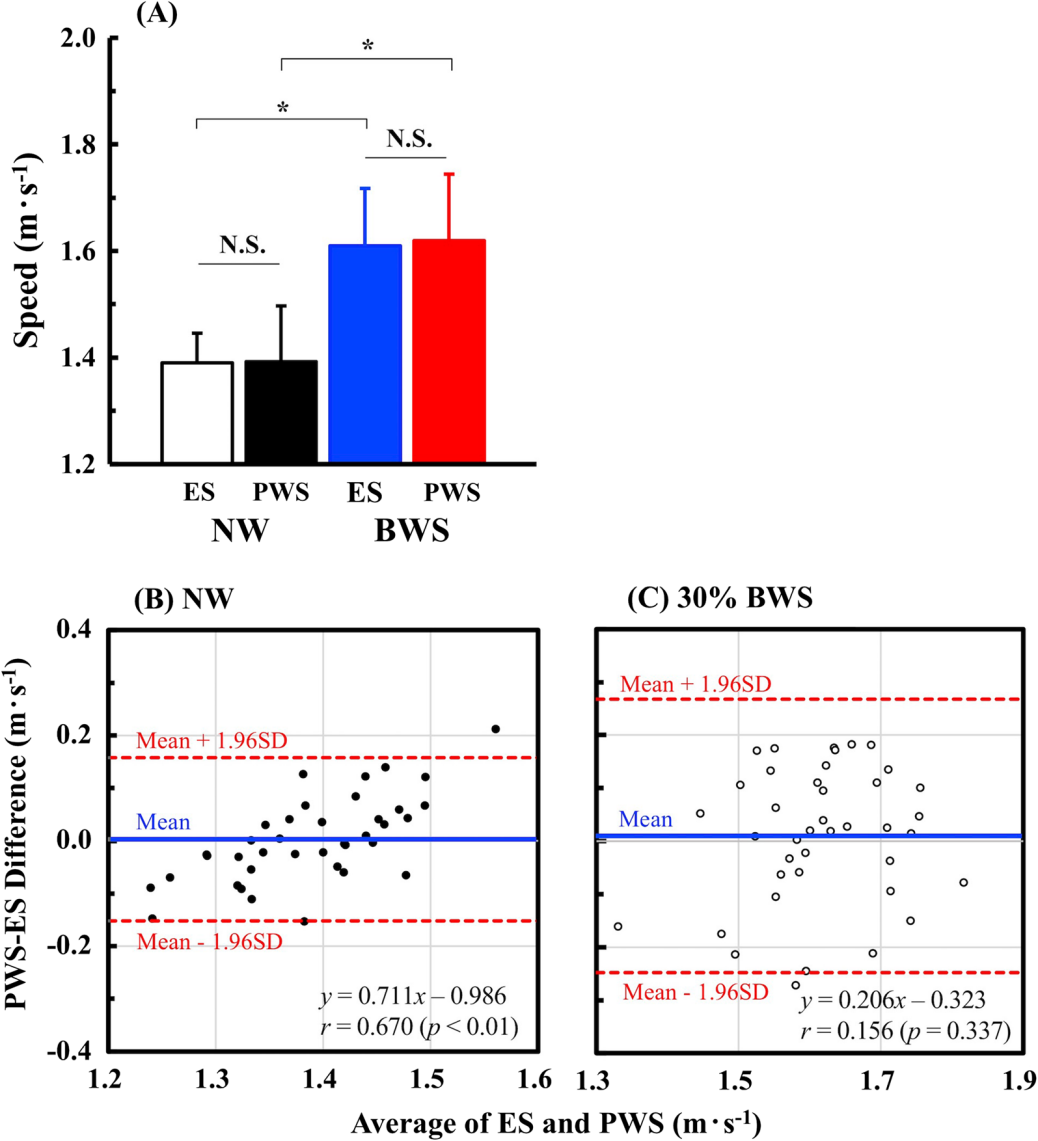
Comparisons of ES and preferred walking speed (PWS). **a** Showed that the ES was not significantly different from the PWS under NW (white and black bars) and BWS (blue and red bars). *p < 0.001 between NW and BWS, and N.S. means non-significant difference between ES and PWS, respectively. Values are mean ± SD. **b** and **c** Showed a Bland-Altman plots to examine the agreement between ES and PWS obtained in the NW and BWS conditions. Blue line indicates the mean of the individual PWS–ES differences. Red broken lines are the upper and lower limits of agreement.

The CoT values were significantly decreased by 9.2% (1.56 m s^− 1^), 15.6% (1.78 m s^− 1^), and 20.8% (2.00 m s^− 1^) with BWS than NW, but not at speeds slower than 1.33 m s^− 1^ (*F* = 7.901, *p* = 0.006; Fig. 2). A significantly faster ES was observed with BWS (1.61 ± 0.11 m s^− 1^) than NW (1.39 ± 0.06 m s^− 1^) (*t* = 11.420, *p* < 0.001; Fig. 2). The PWS was not significantly different from the ES under both conditions (*F* = 0.215, *p* = 0.646; Fig. 3a). From a calculation using the Eq. 4, the CI_95%_ values ranged from − 0.087 to + 0.092 under NW and from − 0.105 to + 0.125 with BWS. The *d*_m_ ± *SD*_d_ values were 0.003 ± 0.079 under NW (Fig. 3b) and 0.010 ± 0.129 with BWS (Fig. 3c). There was a significant relationship between individual PWS–ES difference and individual average of the ES and PWS under NW (*r* = 0.670, *p* < 0.001; Fig. 3b), but not with BWS (*r* = 0.156, *p* = 0.337; Fig. 3c).

## Discussion

The CoT values were significantly lower with BWS than NW at speeds faster than 1.56 m s^− 1^, however, no significantly lower CoT values were observed at speeds slower than 1.33 m s^− 1^ (Fig. 2), resulting in a significantly faster ES with BWS than NW (Fig. 2). These results supported our first hypothesis. However, the metabolic rate during walking at any walking speed has been reported to decrease linearly with increasing body weight support [13, 22–24], and the reduced metabolic rate seemed to depend on walking speed [13, 22–24]. These partial discrepancies between our present study and some previous studies would be derived from different characteristics of used apparatus, such as torso suspension with a long elastic harness [22–24] and lower-body positive pressure [13]. As technical considerations, the faster ES with BWS was potentially associated with a characteristics of a fluctuation of the vertical force caused by spring-like characteristics of our apparatus (Fig. 1b). The stroke length of the spring of our BWS apparatus is much shorter than others [22–24]. When using such an apparatus during walking, the vertical force tends to fluctuate especially at faster speeds (Fig. 1b), because vertical displacement of the center of body mass increases as walking speed increases. At speeds faster than 1.56 m s^− 1^, a percentage of BWS increased by ~ 34% at the propulsive phase of a gait cycle (Fig. 1b), which allows more mechanical work to redirect the center of body mass upward and forward [25]. Reducing the mechanical work at the propulsive phase potentially reduce the metabolic rate or energy cost of walking [26–28], indicating that the reduced CoT values with BWS at speeds faster than 1.56 m s^− 1^ could be attributed to the accelerated increase in the vertical force at the propulsive phase by the spring-like behavior of our BWS apparatus.

In support of our second hypothesis, the PWS was equivalent to the ES not only under the NW but also with BWS (Fig. 3a). No fixed bias was found between ES and PWS in both conditions, because the *d*_m_ was almost zero (blue lines in Fig. 3b, c). Instead, there was a proportional bias between ES and PWS under NW, but not with BWS, because there was a significant relationship between individual PWS–ES difference and individual average of the ES and PWS under NW (*r* = 0.670, *p* < 0.001; Fig. 3b). However, most of the plots under NW was within the upper and lower limits of agreements (Fig. 3b). The CI_95%_ values ranged between negative and positive values in both conditions (Fig. 3b, c), indicating that there was no systematic bias between ES and PWS in both conditions. These statistical results mean that the PWS was almost equivalent to the ES, and the PWS can be a substitutable index of the individual ES, at least, in our participants. A recent longitudinal survey revealed that normal walking speed in daily lives, being essentially linked to the PWS, was already associated with physical and biological functions of accelerating aging even in midlife [29]. PWS can be easily determined in each individual, so that a practical use of the PWS in our daily lives may contribute to monitor our locomotion ability. Other notable intervention studies reported that the PWS was slower on rough terrain compared to smooth terrain, and that the PWS was significantly slower than the ES on both terrains [30]. Visual disturbance also modified the PWS [31]. Therefore, we should acknowledge that the PWS can be modified by several human factors, such as gait instability and visual cognition, through modifications of individual’s optimal combination of stride length and step frequency to minimize the CoT. On the basis of this interpretation, metabolic minimization is presumably the central mechanism to explain the PWS in able-bodied healthy young males.

Study limitations should be acknowledged. First, this study recruited healthy participants only. As explained before, patients after heart surgery or prosthesis users prefer a slower PWS than their 'calculated' ES [3]. However, a use of BWS apparatus can reduce the metabolic rate during walking [13] and running [14, 17] on the treadmill. Given these previous findings and our current results (Fig. 2), a use of BWS apparatus could be effective for training purposes in individuals whose physical fitness is poor. Another potential limitation was that only 30% BWS was tested. As far as we know, a short-stroke spring-like BWS was used only once in the past [17]. In association with the installed spring characteristics, more studies are necessary to understand physiological responses if more and/or less than 30% BW was supported by the spring-type BWS apparatus.

## Conclusion

Reduced CoT values at faster walking speeds made the individual ES faster with 30% BWS compared to the NW. This was due to a spring-like behavior of the BWS apparatus to redirect the body mass upward at faster than 1.56 m s^− 1^. We also found that the ES was equivalent to the PWS in both conditions, suggesting that the PWS could be mainly determined by the metabolic minimization in healthy young males. This result further derives that the PWS can be a substitutable index of the individual ES in these populations.

## Data Availability

The data sets used in this study was are available as supplementary material.

## Abbreviations

ANOVA: analysis of variance
BW: body weight
BWS: body weight support
CI_95%_: confidence interval
CoT: cost of transport
*d*_m_: mean of individual PWS–ES difference
ES: economical speed
NW: normal walking
PWS: preferred walking speed
SD: standard deviation
*SD*_d_: standard deviation of the individual PWS–ES difference
VCO_2_: carbon dioxide output
VO_2_: oxygen uptake
